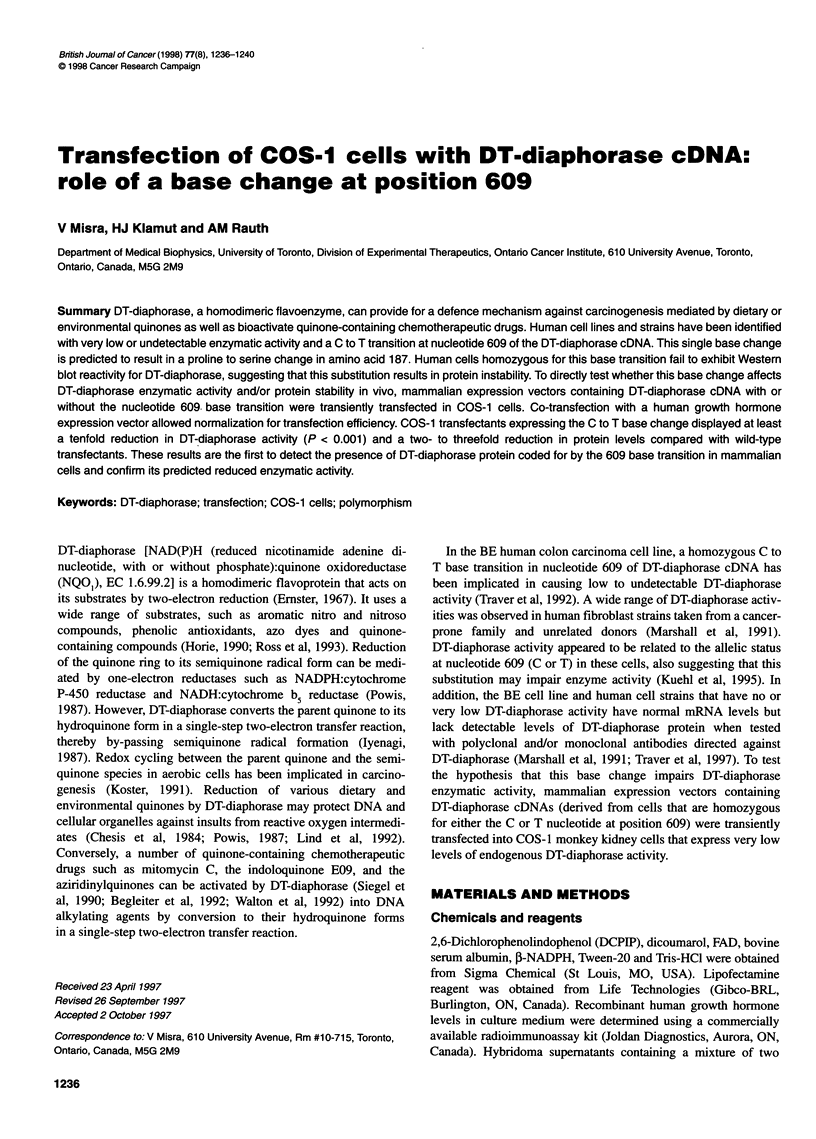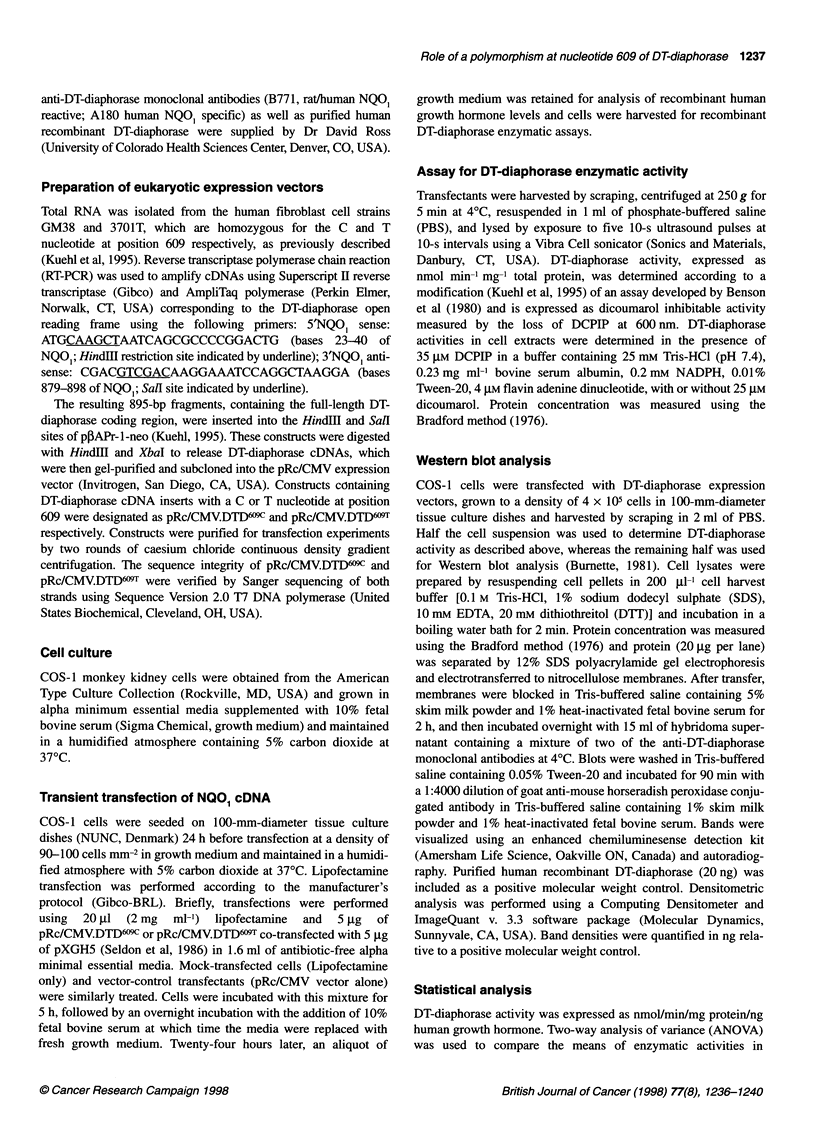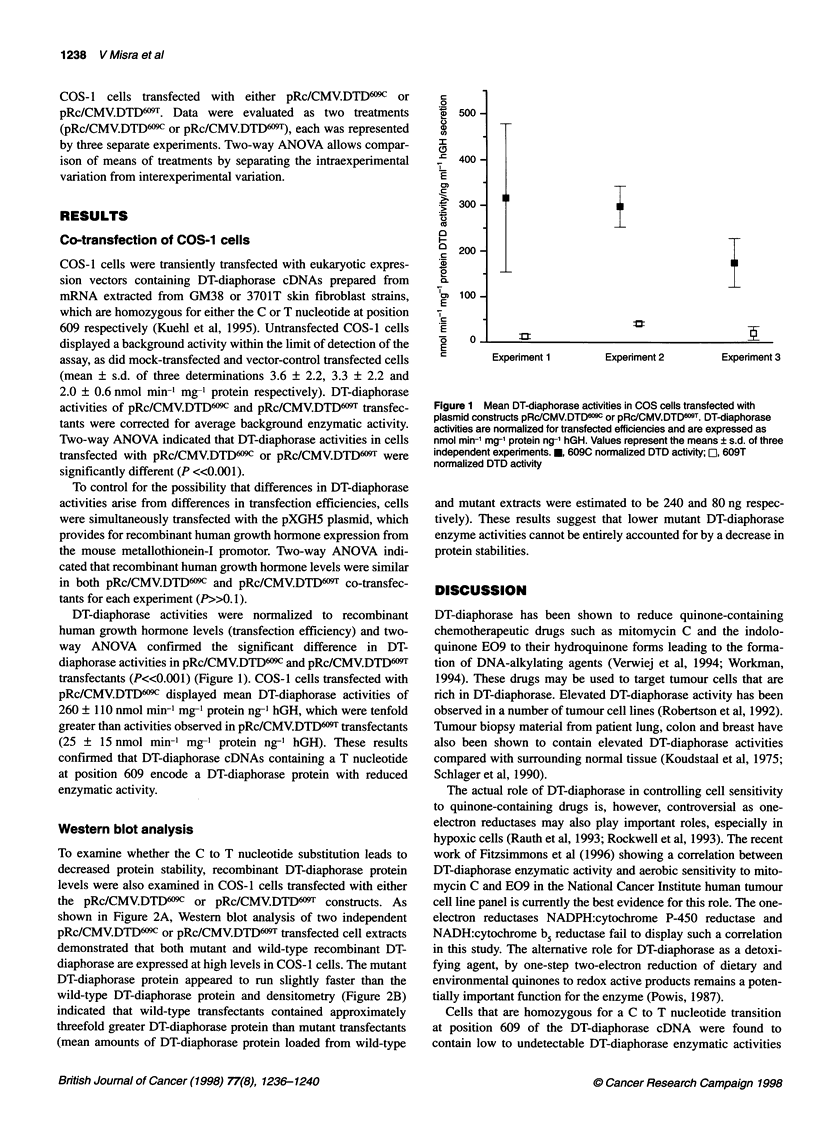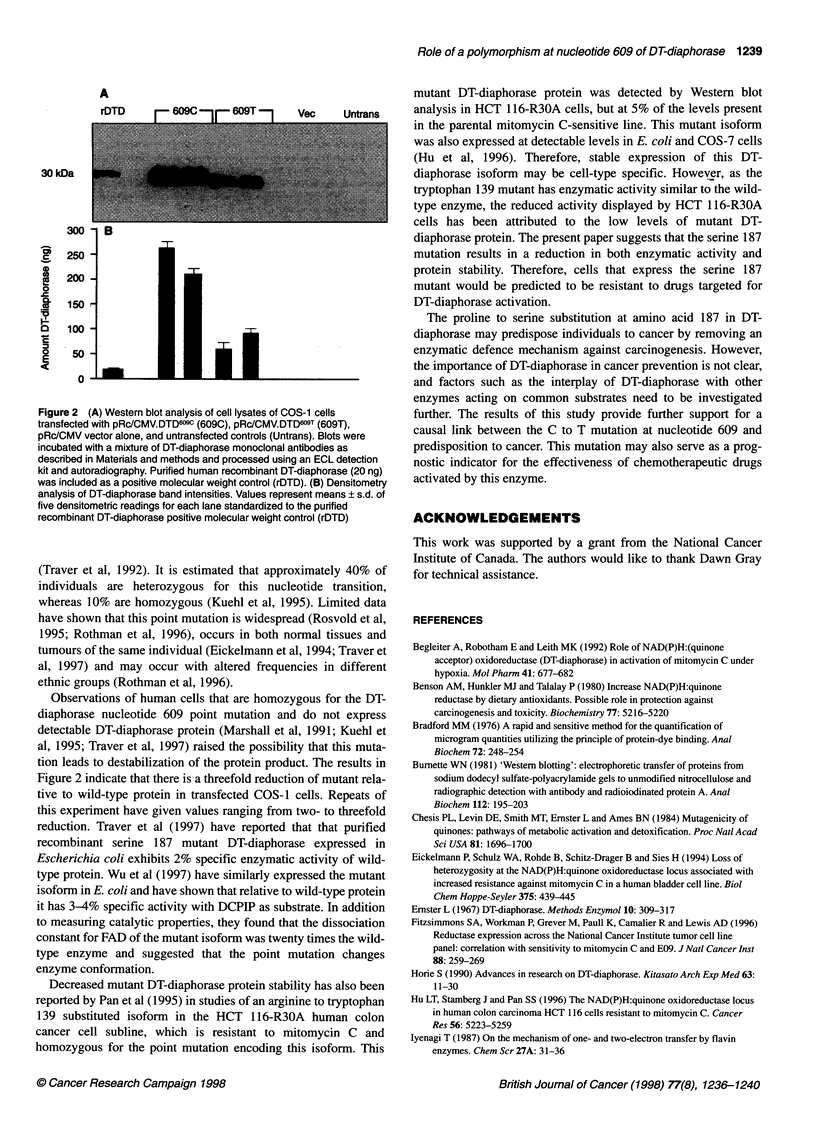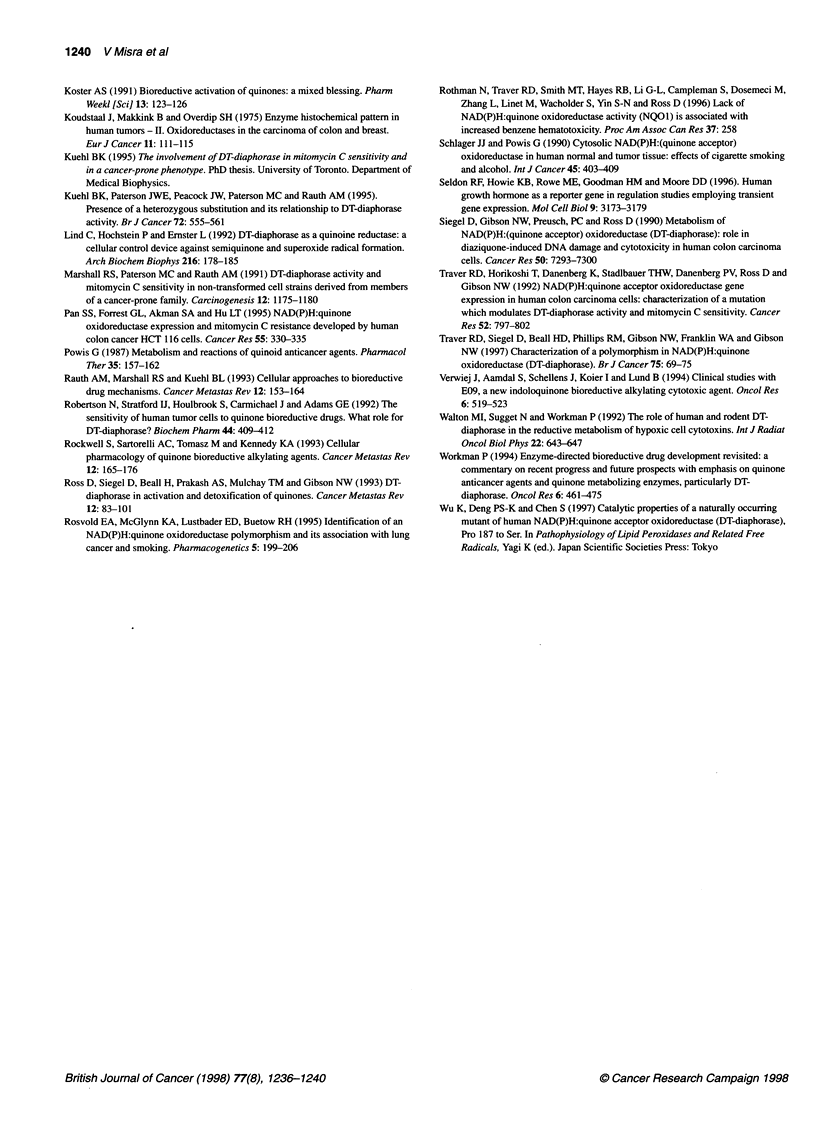# Transfection of COS-1 cells with DT-diaphorase cDNA: role of a base change at position 609.

**DOI:** 10.1038/bjc.1998.208

**Published:** 1998-04

**Authors:** V. Misra, H. J. Klamut, A. M. Rauth

**Affiliations:** Department of Medical Biophysics, University of Toronto, Ontario Cancer Institute, Canada.

## Abstract

DT-diaphorase, a homodimeric flavoenzyme, can provide for a defence mechanism against carcinogenesis mediated by dietary or environmental quinones as well as bioactivate quinone-containing chemotherapeutic drugs. Human cell lines and strains have been identified with very low or undetectable enzymatic activity and a C to T transition at nucleotide 609 of the DT-diaphorase cDNA. This single base change is predicted to result in a proline to serine change in amino acid 187. Human cells homozygous for this base transition fail to exhibit Western blot reactivity for DT-diaphorase, suggesting that this substitution results in protein instability. To directly test whether this base change affects DT-diaphorase enzymatic activity and/or protein stability in vivo, mammalian expression vectors containing DT-diaphorase cDNA with or without the nucleotide 609 base transition were transiently transfected in COS-1 cells. Co-transfection with a human growth hormone expression vector allowed normalization for transfection efficiency. COS-1 transfectants expressing the C to T base change displayed at least a tenfold reduction in DT-diaphorase activity (P < 0.001) and a two- to threefold reduction in protein levels compared with wild-type transfectants. These results are the first to detect the presence of DT-diaphorase protein coded for by the 609 base transition in mammalian cells and confirm its predicted reduced enzymatic activity.


British Journal of Cancer (1998) 77(8), 1236–1240
© 1998 Cancer Research Campaign

### Transfection of COS-1 cells with DT-diaphorase cDNA: role of a base change at position 609

**V Misra, HJ Klamut and AM Rauth**

Department of Medical Biophysics, University of Toronto, Division of Experimental Therapeutics, Ontario Cancer Institute, 610 University Avenue, Toronto, Ontario, Canada, M5G 2M9

**Summary** DT-diaphorase, a homodimeric flavoenzyme, can provide for a defence mechanism against carcinogenesis mediated by dietary or environmental quinones as well as bioactivate quinone-containing chemotherapeutic drugs. Human cell lines and strains have been identified with very low or undetectable enzymatic activity and a C to T transition at nucleotide 609 of the DT-diaphorase cDNA. This single base change is predicted to result in a proline to serine change in amino acid 187. Human cells homozygous for this base transition fail to exhibit Western blot reactivity for DT-diaphorase, suggesting that this substitution results in protein instability. To directly test whether this base change affects DT-diaphorase enzymatic activity and/or protein stability in vivo, mammalian expression vectors containing DT-diaphorase cDNA with or without the nucleotide 609 base transition were transiently transfected in COS-1 cells. Co-transfection with a human growth hormone expression vector allowed normalization for transfection efficiency. COS-1 transfectants expressing the C to T base change displayed at least a tenfold reduction in DT-diaphorase activity ($P < 0.001$) and a two- to threefold reduction in protein levels compared with wild-type transfectants. These results are the first to detect the presence of DT-diaphorase protein coded for by the 609 base transition in mammalian cells and confirm its predicted reduced enzymatic activity.

**Keywords:** DT-diaphorase; transfection; COS-1 cells; polymorphism

DT-diaphorase [NAD(P)H (reduced nicotinamide adenine dinucleotide, with or without phosphate):quinone oxidoreductase ($NQO_1$), EC 1.6.99.2] is a homodimeric flavoprotein that acts on its substrates by two-electron reduction (Ernster, 1967). It uses a wide range of substrates, such as aromatic nitro and nitroso compounds, phenolic antioxidants, azo dyes and quinone-containing compounds (Horie, 1990; Ross et al, 1993). Reduction of the quinone ring to its semiquinone radical form can be mediated by one-electron reductases such as NADPH:cytochrome P-450 reductase and NADH:cytochrome $b_5$ reductase (Powis, 1987). However, DT-diaphorase converts the parent quinone to its hydroquinone form in a single-step two-electron transfer reaction, thereby by-passing semiquinone radical formation (Iyenagi, 1987). Redox cycling between the parent quinone and the semiquinone species in aerobic cells has been implicated in carcinogenesis (Koster, 1991). Reduction of various dietary and environmental quinones by DT-diaphorase may protect DNA and cellular organelles against insults from reactive oxygen intermediates (Chesis et al, 1984; Powis, 1987; Lind et al, 1992). Conversely, a number of quinone-containing chemotherapeutic drugs such as mitomycin C, the indoloquinone E09, and the aziridinylquinones can be activated by DT-diaphorase (Siegel et al, 1990; Begleiter et al, 1992; Walton et al, 1992) into DNA alkylating agents by conversion to their hydroquinone forms in a single-step two-electron transfer reaction.

*Received 23 April 1997*
*Revised 26 September 1997*
*Accepted 2 October 1997*

*Correspondence to:* V Misra, 610 University Avenue, Rm #10-715, Toronto, Ontario, Canada, M5G 2M9

In the BE human colon carcinoma cell line, a homozygous C to T base transition in nucleotide 609 of DT-diaphorase cDNA has been implicated in causing low to undetectable DT-diaphorase activity (Traver et al, 1992). A wide range of DT-diaphorase activities was observed in human fibroblast strains taken from a cancer-prone family and unrelated donors (Marshall et al, 1991). DT-diaphorase activity appeared to be related to the allelic status at nucleotide 609 (C or T) in these cells, also suggesting that this substitution may impair enzyme activity (Kuehl et al, 1995). In addition, the BE cell line and human cell strains that have no or very low DT-diaphorase activity have normal mRNA levels but lack detectable levels of DT-diaphorase protein when tested with polyclonal and/or monoclonal antibodies directed against DT-diaphorase (Marshall et al, 1991; Traver et al, 1997). To test the hypothesis that this base change impairs DT-diaphorase enzymatic activity, mammalian expression vectors containing DT-diaphorase cDNAs (derived from cells that are homozygous for either the C or T nucleotide at position 609) were transiently transfected into COS-1 monkey kidney cells that express very low levels of endogenous DT-diaphorase activity.

### MATERIALS AND METHODS

### Chemicals and reagents

2,6-Dichlorophenolindophenol (DCPIP), dicoumarol, FAD, bovine serum albumin, β-NADPH, Tween-20 and Tris-HCl were obtained from Sigma Chemical (St Louis, MO, USA). Lipofectamine reagent was obtained from Life Technologies (Gibco-BRL, Burlington, ON, Canada). Recombinant human growth hormone levels in culture medium were determined using a commercially available radioimmunoassay kit (Joldan Diagnostics, Aurora, ON, Canada). Hybridoma supernatants containing a mixture of two anti-DT-diaphorase monoclonal antibodies (B771, rat/human $NQO_1$ reactive; A180 human $NQO_1$ specific) as well as purified human recombinant DT-diaphorase were supplied by Dr David Ross (University of Colorado Health Sciences Center, Denver, CO, USA).

### Preparation of eukaryotic expression vectors

Total RNA was isolated from the human fibroblast cell strains GM38 and 3701T, which are homozygous for the C and T nucleotide at position 609 respectively, as previously described (Kuehl et al, 1995). Reverse transcriptase polymerase chain reaction (RT-PCR) was used to amplify cDNAs using Superscript II reverse transcriptase (Gibco) and AmpliTaq polymerase (Perkin Elmer, Norwalk, CT, USA) corresponding to the DT-diaphorase open reading frame using the following primers: 5′$NQO_1$ sense: ATG<u>CAAGCT</u>AATCAGCGCCCCGGACTG (bases 23–40 of $NQO_1$; *Hin*dIII restriction site indicated by underline); 3′$NQO_1$ antisense: CGAC<u>GTCGAC</u>AAGGAAATCCAGGCTAAGGA (bases 879–898 of $NQO_1$; *Sal*I site indicated by underline).

The resulting 895-bp fragments, containing the full-length DT-diaphorase coding region, were inserted into the *Hin*dIII and *Sal*I sites of pβAPr-1-neo (Kuehl, 1995). These constructs were digested with *Hin*dIII and *Xba*I to release DT-diaphorase cDNAs, which were then gel-purified and subcloned into the pRc/CMV expression vector (Invitrogen, San Diego, CA, USA). Constructs containing DT-diaphorase cDNA inserts with a C or T nucleotide at position 609 were designated as pRc/CMV.$DTD^{609C}$ and pRc/CMV.$DTD^{609T}$ respectively. Constructs were purified for transfection experiments by two rounds of caesium chloride continuous density gradient centrifugation. The sequence integrity of pRc/CMV.$DTD^{609C}$ and pRc/CMV.$DTD^{609T}$ were verified by Sanger sequencing of both strands using Sequence Version 2.0 T7 DNA polymerase (United States Biochemical, Cleveland, OH, USA).

### Cell culture

COS-1 monkey kidney cells were obtained from the American Type Culture Collection (Rockville, MD, USA) and grown in alpha minimum essential media supplemented with 10% fetal bovine serum (Sigma Chemical, growth medium) and maintained in a humidified atmosphere containing 5% carbon dioxide at 37°C.

### Transient transfection of $NQO_1$ cDNA

COS-1 cells were seeded on 100-mm-diameter tissue culture dishes (NUNC, Denmark) 24 h before transfection at a density of 90–100 cells $mm^{-2}$ in growth medium and maintained in a humidified atmosphere with 5% carbon dioxide at 37°C. Lipofectamine transfection was performed according to the manufacturer's protocol (Gibco-BRL). Briefly, transfections were performed using 20 µl (2 mg $ml^{-1}$) lipofectamine and 5 µg of pRc/CMV.$DTD^{609C}$ or pRc/CMV.$DTD^{609T}$ co-transfected with 5 µg of pXGH5 (Seldon et al, 1986) in 1.6 ml of antibiotic-free alpha minimal essential media. Mock-transfected cells (Lipofectamine only) and vector-control transfectants (pRc/CMV vector alone) were similarly treated. Cells were incubated with this mixture for 5 h, followed by an overnight incubation with the addition of 10% fetal bovine serum at which time the media were replaced with fresh growth medium. Twenty-four hours later, an aliquot of growth medium was retained for analysis of recombinant human growth hormone levels and cells were harvested for recombinant DT-diaphorase enzymatic assays.

### Assay for DT-diaphorase enzymatic activity

Transfectants were harvested by scraping, centrifuged at 250 ***g*** for 5 min at 4°C, resuspended in 1 ml of phosphate-buffered saline (PBS), and lysed by exposure to five 10-s ultrasound pulses at 10-s intervals using a Vibra Cell sonicator (Sonics and Materials, Danbury, CT, USA). DT-diaphorase activity, expressed as nmol $min^{-1}$ $mg^{-1}$ total protein, was determined according to a modification (Kuehl et al, 1995) of an assay developed by Benson et al (1980) and is expressed as dicoumarol inhibitable activity measured by the loss of DCPIP at 600 nm. DT-diaphorase activities in cell extracts were determined in the presence of 35 µM DCPIP in a buffer containing 25 mM Tris-HCl (pH 7.4), 0.23 mg $ml^{-1}$ bovine serum albumin, 0.2 mM NADPH, 0.01% Tween-20, 4 µM flavin adenine dinucleotide, with or without 25 µM dicoumarol. Protein concentration was measured using the Bradford method (1976).

### Western blot analysis

COS-1 cells were transfected with DT-diaphorase expression vectors, grown to a density of $4 \times 10^5$ cells in 100-mm-diameter tissue culture dishes and harvested by scraping in 2 ml of PBS. Half the cell suspension was used to determine DT-diaphorase activity as described above, whereas the remaining half was used for Western blot analysis (Burnette, 1981). Cell lysates were prepared by resuspending cell pellets in 200 $\mu l^{-1}$ cell harvest buffer [0.1 M Tris-HCl, 1% sodium dodecyl sulphate (SDS), 10 mM EDTA, 20 mM dithiothreitol (DTT)] and incubation in a boiling water bath for 2 min. Protein concentration was measured using the Bradford method (1976) and protein (20 µg per lane) was separated by 12% SDS polyacrylamide gel electrophoresis and electrotransferred to nitrocellulose membranes. After transfer, membranes were blocked in Tris-buffered saline containing 5% skim milk powder and 1% heat-inactivated fetal bovine serum for 2 h, and then incubated overnight with 15 ml of hybridoma supernatant containing a mixture of two of the anti-DT-diaphorase monoclonal antibodies at 4°C. Blots were washed in Tris-buffered saline containing 0.05% Tween-20 and incubated for 90 min with a 1:4000 dilution of goat anti-mouse horseradish peroxidase conjugated antibody in Tris-buffered saline containing 1% skim milk powder and 1% heat-inactivated fetal bovine serum. Bands were visualized using an enhanced chemiluminesense detection kit (Amersham Life Science, Oakville ON, Canada) and autoradiography. Purified human recombinant DT-diaphorase (20 ng) was included as a positive molecular weight control. Densitometric analysis was performed using a Computing Densitometer and ImageQuant v. 3.3 software package (Molecular Dynamics, Sunnyvale, CA, USA). Band densities were quantified in ng relative to a positive molecular weight control.

### Statistical analysis

DT-diaphorase activity was expressed as nmol/min/mg protein/ng human growth hormone. Two-way analysis of variance (ANOVA) was used to compare the means of enzymatic activities in

© Cancer Research Campaign 1998

COS-1 cells transfected with either pRc/CMV.DTD$^{609C}$ or pRc/CMV.DTD$^{609T}$. Data were evaluated as two treatments (pRc/CMV.DTD$^{609C}$ or pRc/CMV.DTD$^{609T}$), each was represented by three separate experiments. Two-way ANOVA allows comparison of means of treatments by separating the intraexperimental variation from interexperimental variation.

### RESULTS

### Co-transfection of COS-1 cells

COS-1 cells were transiently transfected with eukaryotic expression vectors containing DT-diaphorase cDNAs prepared from mRNA extracted from GM38 or 3701T skin fibroblast strains, which are homozygous for either the C or T nucleotide at position 609 respectively (Kuehl et al, 1995). Untransfected COS-1 cells displayed a background activity within the limit of detection of the assay, as did mock-transfected and vector-control transfected cells (mean ± s.d. of three determinations 3.6 ± 2.2, 3.3 ± 2.2 and 2.0 ± 0.6 nmol min$^{-1}$ mg$^{-1}$ protein respectively). DT-diaphorase activities of pRc/CMV.DTD$^{609C}$ and pRc/CMV.DTD$^{609T}$ transfectants were corrected for average background enzymatic activity. Two-way ANOVA indicated that DT-diaphorase activities in cells transfected with pRc/CMV.DTD$^{609C}$ or pRc/CMV.DTD$^{609T}$ were significantly different ($P \ll 0.001$).

To control for the possibility that differences in DT-diaphorase activities arise from differences in transfection efficiencies, cells were simultaneously transfected with the pXGH5 plasmid, which provides for recombinant human growth hormone expression from the mouse metallothionein-I promotor. Two-way ANOVA indicated that recombinant human growth hormone levels were similar in both pRc/CMV.DTD$^{609C}$ and pRc/CMV.DTD$^{609T}$ co-transfectants for each experiment ($P \gg 0.1$).

DT-diaphorase activities were normalized to recombinant human growth hormone levels (transfection efficiency) and two-way ANOVA confirmed the significant difference in DT-diaphorase activities in pRc/CMV.DTD$^{609C}$ and pRc/CMV.DTD$^{609T}$ transfectants ($P \ll 0.001$) (Figure 1). COS-1 cells transfected with pRc/CMV.DTD$^{609C}$ displayed mean DT-diaphorase activities of 260 ± 110 nmol min$^{-1}$ mg$^{-1}$ protein ng$^{-1}$ hGH, which were tenfold greater than activities observed in pRc/CMV.DTD$^{609T}$ transfectants (25 ± 15 nmol min$^{-1}$ mg$^{-1}$ protein ng$^{-1}$ hGH). These results confirmed that DT-diaphorase cDNAs containing a T nucleotide at position 609 encode a DT-diaphorase protein with reduced enzymatic activity.

### Western blot analysis

To examine whether the C to T nucleotide substitution leads to decreased protein stability, recombinant DT-diaphorase protein levels were also examined in COS-1 cells transfected with either the pRc/CMV.DTD$^{609C}$ or pRc/CMV.DTD$^{609T}$ constructs. As shown in Figure 2A, Western blot analysis of two independent pRc/CMV.DTD$^{609C}$ or pRc/CMV.DTD$^{609T}$ transfected cell extracts demonstrated that both mutant and wild-type recombinant DT-diaphorase are expressed at high levels in COS-1 cells. The mutant DT-diaphorase protein appeared to run slightly faster than the wild-type DT-diaphorase protein and densitometry (Figure 2B) indicated that wild-type transfectants contained approximately threefold greater DT-diaphorase protein than mutant transfectants (mean amounts of DT-diaphorase protein loaded from wild-type

**Figure 1** Mean DT-diaphorase activities in COS cells transfected with plasmid constructs pRc/CMV.DTD$^{609C}$ or pRc/CMV.DTD$^{609T}$. DT-diaphorase activities are normalized for transfected efficiencies and are expressed as nmol min$^{-1}$ mg$^{-1}$ protein ng$^{-1}$ hGH. Values represent the means ± s.d. of three independent experiments. ■, 609C normalized DTD activity; □, 609T normalized DTD activity and mutant extracts were estimated to be 240 and 80 ng respectively). These results suggest that lower mutant DT-diaphorase enzyme activities cannot be entirely accounted for by a decrease in protein stabilities.

### DISCUSSION

DT-diaphorase has been shown to reduce quinone-containing chemotherapeutic drugs such as mitomycin C and the indoloquinone EO9 to their hydroquinone forms leading to the formation of DNA-alkylating agents (Verwiej et al, 1994; Workman, 1994). These drugs may be used to target tumour cells that are rich in DT-diaphorase. Elevated DT-diaphorase activity has been observed in a number of tumour cell lines (Robertson et al, 1992). Tumour biopsy material from patient lung, colon and breast have also been shown to contain elevated DT-diaphorase activities compared with surrounding normal tissue (Koudstaal et al, 1975; Schlager et al, 1990).

The actual role of DT-diaphorase in controlling cell sensitivity to quinone-containing drugs is, however, controversial as one-electron reductases may also play important roles, especially in hypoxic cells (Rauth et al, 1993; Rockwell et al, 1993). The recent work of Fitzsimmons et al (1996) showing a correlation between DT-diaphorase enzymatic activity and aerobic sensitivity to mitomycin C and EO9 in the National Cancer Institute human tumour cell line panel is currently the best evidence for this role. The one-electron reductases NADPH:cytochrome P-450 reductase and NADH:cytochrome $b_5$ reductase fail to display such a correlation in this study. The alternative role for DT-diaphorase as a detoxifying agent, by one-step two-electron reduction of dietary and environmental quinones to redox active products remains a potentially important function for the enzyme (Powis, 1987).

Cells that are homozygous for a C to T nucleotide transition at position 609 of the DT-diaphorase cDNA were found to contain low to undetectable DT-diaphorase enzymatic activities

 © Cancer Research Campaign 1998

**Figure 2** (**A**) Western blot analysis of cell lysates of COS-1 cells transfected with pRc/CMV.DTD$^{609C}$ (609C), pRc/CMV.DTD$^{609T}$ (609T), pRc/CMV vector alone, and untransfected controls (Untrans). Blots were incubated with a mixture of DT-diaphorase monoclonal antibodies as described in Materials and methods and processed using an ECL detection kit and autoradiography. Purified human recombinant DT-diaphorase (20 ng) was included as a positive molecular weight control (rDTD). (**B**) Densitometry analysis of DT-diaphorase band intensities. Values represent means ± s.d. of five densitometric readings for each lane standardized to the purified recombinant DT-diaphorase positive molecular weight control (rDTD)

(Traver et al, 1992). It is estimated that approximately 40% of individuals are heterozygous for this nucleotide transition, whereas 10% are homozygous (Kuehl et al, 1995). Limited data have shown that this point mutation is widespread (Rosvold et al, 1995; Rothman et al, 1996), occurs in both normal tissues and tumours of the same individual (Eickelmann et al, 1994; Traver et al, 1997) and may occur with altered frequencies in different ethnic groups (Rothman et al, 1996).

Observations of human cells that are homozygous for the DT-diaphorase nucleotide 609 point mutation and do not express detectable DT-diaphorase protein (Marshall et al, 1991; Kuehl et al, 1995; Traver et al, 1997) raised the possibility that this mutation leads to destabilization of the protein product. The results in Figure 2 indicate that there is a threefold reduction of mutant relative to wild-type protein in transfected COS-1 cells. Repeats of this experiment have given values ranging from two- to threefold reduction. Traver et al (1997) have reported that that purified recombinant serine 187 mutant DT-diaphorase expressed in *Escherichia coli* exhibits 2% specific enzymatic activity of wild-type protein. Wu et al (1997) have similarly expressed the mutant isoform in *E. coli* and have shown that relative to wild-type protein it has 3–4% specific activity with DCPIP as substrate. In addition to measuring catalytic properties, they found that the dissociation constant for FAD of the mutant isoform was twenty times the wild-type enzyme and suggested that the point mutation changes enzyme conformation.

Decreased mutant DT-diaphorase protein stability has also been reported by Pan et al (1995) in studies of an arginine to tryptophan 139 substituted isoform in the HCT 116-R30A human colon cancer cell subline, which is resistant to mitomycin C and homozygous for the point mutation encoding this isoform. This mutant DT-diaphorase protein was detected by Western blot analysis in HCT 116-R30A cells, but at 5% of the levels present in the parental mitomycin C-sensitive line. This mutant isoform was also expressed at detectable levels in *E. coli* and COS-7 cells (Hu et al, 1996). Therefore, stable expression of this DT-diaphorase isoform may be cell-type specific. However, as the tryptophan 139 mutant has enzymatic activity similar to the wild-type enzyme, the reduced activity displayed by HCT 116-R30A cells has been attributed to the low levels of mutant DT-diaphorase protein. The present paper suggests that the serine 187 mutation results in a reduction in both enzymatic activity and protein stability. Therefore, cells that express the serine 187 mutant would be predicted to be resistant to drugs targeted for DT-diaphorase activation.

The proline to serine substitution at amino acid 187 in DT-diaphorase may predispose individuals to cancer by removing an enzymatic defence mechanism against carcinogenesis. However, the importance of DT-diaphorase in cancer prevention is not clear, and factors such as the interplay of DT-diaphorase with other enzymes acting on common substrates need to be investigated further. The results of this study provide further support for a causal link between the C to T mutation at nucleotide 609 and predisposition to cancer. This mutation may also serve as a prognostic indicator for the effectiveness of chemotherapeutic drugs activated by this enzyme.

### ACKNOWLEDGEMENTS

This work was supported by a grant from the National Cancer Institute of Canada. The authors would like to thank Dawn Gray for technical assistance.

### REFERENCES

Begleiter A, Robotham E and Leith MK (1992) Role of NAD(P)H:(quinone acceptor) oxidoreductase (DT-diaphorase) in activation of mitomycin C under hypoxia. *Mol Pharm* **41**: 677–682

Benson AM, Hunkler MJ and Talalay P (1980) Increase NAD(P)H:quinone reductase by dietary antioxidants. Possible role in protection against carcinogenesis and toxicity. *Biochemistry* **77**: 5216–5220

Bradford MM (1976) A rapid and sensitive method for the quantification of microgram quantities utilizing the principle of protein-dye binding. *Anal Biochem* **72**: 248–254

Burnette WN (1981) 'Western blotting': electrophoretic transfer of proteins from sodium dodecyl sulfate-polyacrylamide gels to unmodified nitrocellulose and radiographic detection with antibody and radioiodinated protein A. *Anal Biochem* **112**: 195–203

Chesis PL, Levin DE, Smith MT, Ernster L and Ames BN (1984) Mutagenicity of quinones: pathways of metabolic activation and detoxification. *Proc Natl Acad Sci USA* **81**: 1696–1700

Eickelmann P, Schulz WA, Rohde B, Schitz-Drager B and Sies H (1994) Loss of heterozygosity at the NAD(P)H:quinone oxidoreductase locus associated with increased resistance against mitomycin C in a human bladder cell line. *Biol Chem Hoppe-Seyler* **375**: 439–445

Ernster L (1967) DT-diaphorase. *Methods Enzymol* **10**: 309–317

Fitzsimmons SA, Workman P, Grever M, Paull K, Camalier R and Lewis AD (1996) Reductase expression across the National Cancer Institute tumor cell line panel: correlation with sensitivity to mitomycin C and E09. *J Natl Cancer Inst* **88**: 259–269

Horie S (1990) Advances in research on DT-diaphorase. *Kitasato Arch Exp Med* **63**: 11–30

Hu LT, Stamberg J and Pan SS (1996) The NAD(P)H:quinone oxidoreductase locus in human colon carcinoma HCT 116 cells resistant to mitomycin C. *Cancer Res* **56**: 5223–5259

Iyenagi T (1987) On the mechanism of one- and two-electron transfer by flavin enzymes. *Chem Scr* **27A**: 31–36

© Cancer Research Campaign 1998 

Koster AS (1991) Bioreductive activation of quinones: a mixed blessing. *Pharm Weekl [Sci]* **13**: 123–126

Koudstaal J, Makkink B and Overdip SH (1975) Enzyme histochemical pattern in human tumors – II. Oxidoreductases in the carcinoma of colon and breast. *Eur J Cancer* **11**: 111–115

Kuehl BK (1995) *The involvement of DT-diaphorase in mitomycin C sensitivity and in a cancer-prone phenotype*. PhD thesis. University of Toronto. Department of Medical Biophysics.

Kuehl BK, Paterson JWE, Peacock JW, Paterson MC and Rauth AM (1995). Presence of a heterozygous substitution and its relationship to DT-diaphorase activity. *Br J Cancer* **72**: 555–561

Lind C, Hochstein P and Ernster L (1992) DT-diaphorase as a quinoine reductase: a cellular control device against semiquinone and superoxide radical formation. *Arch Biochem Biophys* **216**: 178–185

Marshall RS, Paterson MC and Rauth AM (1991) DT-diaphorase activity and mitomycin C sensitivity in non-transformed cell strains derived from members of a cancer-prone family. *Carcinogenesis* **12**: 1175–1180

Pan SS, Forrest GL, Akman SA and Hu LT (1995) NAD(P)H:quinone oxidoreductase expression and mitomycin C resistance developed by human colon cancer HCT 116 cells. *Cancer Res* **55**: 330–335

Powis G (1987) Metabolism and reactions of quinoid anticancer agents. *Pharmacol Ther* **35**: 157–162

Rauth AM, Marshall RS and Kuehl BL (1993) Cellular approaches to bioreductive drug mechanisms. *Cancer Metastas Rev* **12**: 153–164

Robertson N, Stratford IJ, Houlbrook S, Carmichael J and Adams GE (1992) The sensitivity of human tumor cells to quinone bioreductive drugs. What role for DT-diaphorase? *Biochem Pharm* **44**: 409–412

Rockwell S, Sartorelli AC, Tomasz M and Kennedy KA (1993) Cellular pharmacology of quinone bioreductive alkylating agents. *Cancer Metastas Rev* **12**: 165–176

Ross D, Siegel D, Beall H, Prakash AS, Mulchay TM and Gibson NW (1993) DT-diaphorase in activation and detoxification of quinones. *Cancer Metastas Rev* **12**: 83–101

Rosvold EA, McGlynn KA, Lustbader ED, Buetow RH (1995) Identification of an NAD(P)H:quinone oxidoreductase polymorphism and its association with lung cancer and smoking. *Pharmacogenetics* **5**: 199–206

Rothman N, Traver RD, Smith MT, Hayes RB, Li G-L, Campleman S, Dosemeci M, Zhang L, Linet M, Wacholder S, Yin S-N and Ross D (1996) Lack of NAD(P)H:quinone oxidoreductase activity (NQO1) is associated with increased benzene hematotoxicity. *Proc Am Assoc Can Res* **37**: 258

Schlager JJ and Powis G (1990) Cytosolic NAD(P)H:(quinone acceptor) oxidoreductase in human normal and tumor tissue: effects of cigarette smoking and alcohol. *Int J Cancer* **45**: 403–409

Seldon RF, Howie KB, Rowe ME, Goodman HM and Moore DD (1996). Human growth hormone as a reporter gene in regulation studies employing transient gene expression. *Mol Cell Biol* **9**: 3173–3179

Siegel D, Gibson NW, Preusch, PC and Ross D (1990) Metabolism of NAD(P)H:(quinone acceptor) oxidoreductase (DT-diaphorase): role in diaziquone-induced DNA damage and cytotoxicity in human colon carcinoma cells. *Cancer Res* **50**: 7293–7300

Traver RD, Horikoshi T, Danenberg K, Stadlbauer THW, Danenberg PV, Ross D and Gibson NW (1992) NAD(P)H:quinone acceptor oxidoreductase gene expression in human colon carcinoma cells: characterization of a mutation which modulates DT-diaphorase activity and mitomycin C sensitivity. *Cancer Res* **52**: 797–802

Traver RD, Siegel D, Beall HD, Phillips RM, Gibson NW, Franklin WA and Gibson NW (1997) Characterization of a polymorphism in NAD(P)H:quinone oxidoreductase (DT-diaphorase). *Br J Cancer* **75**: 69–75

Verwiej J, Aamdal S, Schellens J, Koier I and Lund B (1994) Clinical studies with E09, a new indoloquinone bioreductive alkylating cytotoxic agent. *Oncol Res* **6**: 519–523

Walton MI, Sugget N and Workman P (1992) The role of human and rodent DT-diaphorase in the reductive metabolism of hypoxic cell cytotoxins. *Int J Radiat Oncol Biol Phys* **22**: 643–647

Workman P (1994) Enzyme-directed bioreductive drug development revisited: a commentary on recent progress and future prospects with emphasis on quinone anticancer agents and quinone metabolizing enzymes, particularly DT-diaphorase. *Oncol Res* **6**: 461–475

Wu K, Deng PS-K and Chen S (1997) Catalytic properties of a naturally occurring mutant of human NAD(P)H:quinone acceptor oxidoreductase (DT-diaphorase), Pro 187 to Ser. In *Pathophysiology of Lipid Peroxidases and Related Free Radicals,* Yagi K (ed.). Japan Scientific Societies Press: Tokyo

© Cancer Research Campaign 1998